# BACE1 expression in neurodegenerative and neurodevelopmental diseases

**DOI:** 10.1002/alz.090694

**Published:** 2025-01-09

**Authors:** Leda Leme Talib, Augusto Magno Tranquezi Cordeiro, Wagner Farid Gattaz, Orestes Vicente Forlenza

**Affiliations:** ^1^ Laboratory of Neurosciences (LIM27), Departamento e Instituto de Psiquiatria, Hospital das Clínicas, Faculdade de Medicina da Universidade de São Paulo, São Paulo Brazil; ^2^ Instituto de Psiquiatria do Hospital das Clínicas da Faculdade de Medicina da Universidade de São Paulo, São Paulo, São Paulo Brazil; ^3^ University of São Paulo, São Paulo, Brazil, Sao Paulo, São Paulo Brazil; ^4^ Laboratory of Neuroscience (LIM27), Departamento e Instituto de Psiquiatria, Hospital das Clínicas, Faculdade de Medicina da Universidade de São Paulo, São Paulo, São Paulo Brazil

## Abstract

**Background:**

Beta‐site amyloid precursor protein cleaving enzyme 1 (BACE1) was first identified as the rate limiting enzyme of amyloid‐β‐peptide (Aβ) production. The catalytic activity of BACE1 favors the generation of Aβ peptides and overproduction and accumulation of Aβ in the brain triggers downstream neurotoxic events that pertain to the amyloid cascade, leading to the formation of neuritic plaques. Furthermore BACE1 acts in the synapse through processing substrates such as APP‐like proteins, Neuregulin‐1 (Nrg 1), and β2 and β4 subunits of voltage‐gated Na+ channels. Nrg1 is a major physiological substrate of BACE1 during early postnatal development and one of the most important proteins in myelin formation. The cleavage of Nrg1 by BACE1 is essential for synapse development, cell survival and remyelination. However, BACE1 knockout animals have many subtle phenotypic alterations, including deficits in long‐term potentiation, increased seizure susceptibility, and schizophrenic behavior. It is well known that elderly individuals with Down syndrome (SD) develop amyloid plaques similar to those found in Alzheimer's disease (AD).

**Method:**

Taking this into account our objective was to confirm the BACE1 role in neurodegenerative diseases (23 AD), in 46 DS and 51 individuals with schizophenia (SCZ) and compare them to 79 healthy controls (HC). The enzyme BACE1 expression were determined by western blotting.

**Result:**

The Kruskal‐wallis analysis show that AD patients present an increase in BACE1 in relation to the other groups (HC, DS and SCZ) (p<0.001). The DSCF pairwise comparasion show that SCZ present a decrease in BACE1 expression compared to HC (p=<0.001). SD present no diference related to HC and SCZ (p=0.515 and p=0.09 respectively) and no differences were observed in BACE1 between DS with cognitive decline and without cognitive decline. As well as no difference between SCZ resistant or not to treatment were noted.

**Conclusion:**

Our hypothesis is that the amyloidogenesis observed in DS is not derived from the proteolytic action of BACE1 and that possibly the phisiological process of DS is closer to a neurodevelopmental disease as SCZ.

